# Stacking three late blight resistance genes from wild species directly into African highland potato varieties confers complete field resistance to local blight races

**DOI:** 10.1111/pbi.13042

**Published:** 2018-12-21

**Authors:** Marc Ghislain, Arinaitwe Abel Byarugaba, Eric Magembe, Anne Njoroge, Cristina Rivera, María Lupe Román, José Carlos Tovar, Soledad Gamboa, Gregory A. Forbes, Jan F. Kreuze, Alex Barekye, Andrew Kiggundu

**Affiliations:** ^1^ International Potato Center Nairobi Kenya; ^2^ Kachwekano Zonal Agricultural Research and Development Institute Kabale Uganda; ^3^ International Potato Center Lima Peru; ^4^ National Agriculture Research Laboratories (NARL) Kampala Uganda; ^5^ Present address: Universidad Nacional Agraria La Molina Lima 12 Peru; ^6^ Present address: Donald Danforth Plant Science Center 975 North Warson Road St. Louis Missouri 63132 USA

**Keywords:** GM potatoes, transformation, late blight resistance, *Phytophthora infestans*

## Abstract

Considered responsible for one million deaths in Ireland and widespread famine in the European continent during the 1840s, late blight, caused by *Phytophthora infestans*, remains the most devastating disease of potato (*Solanum tuberosum* L.) with about 15%–30% annual yield loss in sub‐Saharan Africa, affecting mainly smallholder farmers. We show here that the transfer of three resistance (*R*) genes from wild relatives [*RB*,* Rpi‐blb2* from *Solanum bulbocastanum* and *Rpi‐vnt1.1* from *S. venturii*] into potato provided complete resistance in the field over several seasons. We observed that the stacking of the three *R* genes produced a high frequency of transgenic events with resistance to late blight. In the field, 13 resistant transgenic events with the 3*R*‐gene stack from the potato varieties ‘Desiree’ and ‘Victoria’ grew normally without showing pathogen damage and without any fungicide spray, whereas their non‐transgenic equivalent varieties were rapidly killed. Characteristics of the local pathogen population suggest that the resistance to late blight may be long‐lasting because it has low diversity, and essentially consists of the single lineage, 2_A1, which expresses the cognate avirulence effector genes. Yields of two transgenic events from ‘Desiree’ and ‘Victoria’ grown without fungicide to reflect small‐scale farm holders were estimated to be 29 and 45 t/ha respectively. This represents a three to four‐fold increase over the national average. Thus, these late blight resistant potato varieties, which are the farmers’ preferred varieties, could be rapidly adopted and bring significant income to smallholder farmers in sub‐Saharan Africa.

## Introduction

In most areas where potatoes grow, late blight (LB), caused by the oomycete *Phytophthora infestans,* will devastate the crop unless it is protected by fungicides, with as many as 15 sprays per season. The Irish potato famine was in part caused by LB's sudden destruction of potato production which contributed to the loss of at least two million lives 180 years ago in Europe. In developing countries, the affected area is currently between two and three million hectares (Mha) out of 13 Mha of the world potato cultivated area (Haverkort *et al*., 2009). The conservative estimates of 15% loss on actual yield and of 25% on attainable yield due to LB exceed 6.7 to 15 billion dollar losses annually (Haverkort *et al*., 2009). In Uganda, LB has been reported to cause yield losses between 13% and 57% of the total production of potato, which is 770 000 tons (Hareau *et al*., 2014; Kakuhenzire, 2009). Considering an average Ugandan potato farmer growing potatoes on 0.25 ha, the savings on fungicide use and the increase in production represents an additional income of at least $150 per year, which is a significant improvement. Other benefits would also derive, including simplified production management and reduced exposure of farmers and their families to toxic chemicals (Crissman *et al*., 1994). Hence, the replacement of the existing LB susceptible varieties by resistant ones would radically change the lives of many potato growers by increasing incomes and reducing health risks due to exposure to fungicides.

The LB pathogen population in sub‐Saharan Africa (SSA) is remarkably uniform. Until 2007, isolates of *P. infestans* from SSA countries were grouped mainly into one single ‘old’ lineage, US‐1, with few of them pertaining to a ‘new’ lineage, 2_A1, previously referred to as KE‐1 (Pule *et al*., 2013). Being both of the A1 mating type, no sexual recombination occurred to diversify the population. Since 2007, the 2_A1 lineage, more recent and less diverse, has been displacing the US‐1 with rare detection of both lineages in the same location in SSA (Njoroge *et al*., 2016). This narrow pathogen diversity in SSA countries is likely due to the limited exchange of seed potato from countries where pathogen diversity is much higher, as in Europe or North America.

Potato varieties currently grown in Africa and the rest of the world are highly to moderately susceptible to LB and the control of this disease relies exclusively on multiple fungicide applications which select over time for *P. infestans* strains that are resistant to certain classes of fungicide. The most sustainable and environmentally sound way to control LB is by incorporating natural resistance into potato cultivars. Traditional breeding has had limited success in developing varieties with durable resistance to LB. Breeders have used single *R* genes from the wild species *Solanum demissum* but these have been rapidly overcome by new strains of the pathogen. It took about 45 years to release two LB resistant varieties, ‘Bionica’ and ‘Toluca’, bearing *R* gene(s) and other defence genes from the original interspecific hybridization with *S. bulbocastanum* (Haverkort *et al*., 2009). The introgression through conventional breeding of two or three *R* genes from different wild species would take several decades of crossing and selection due to the genetic drag of negative alleles from wild species which is difficult to eliminate in an out‐breeding tetraploid crop.

Among the resistance and defence systems of the plant, effector‐triggered‐immunity (ETI) is particularly effective at controlling pathogens with a biotrophic phase such as *P. infestans*. ETI resistance genes are extremely powerful as the proteins produced by *R* genes recognize, often directly, proteins secreted by the pathogen avirulence (*Avr*) genes and are thus extremely specific and mobilize limited resources from the plant, unlike other pathogen defence systems which may pose a burden on yield (Ning *et al*., 2017). These *R* genes are ubiquitous in plants and code for the nucleotide‐binding domain and the leucine‐rich repeat containing proteins (NB‐LRR) which perceive a wide range of pathogens using diverse mechanistic and structural features (Cesari, 2017). However, the durability of such resistance depends on how essential the effector for pathogenicity is to the pathogen, the pathogen's ability to suppress host immunity, pathogen population diversity, and how many ETI resistance genes are in play. *P. infestans* has hundreds of effectors in its genome which potentially represent a certain level of redundancy in their contribution to pathogenicity (Birch *et al*., 2008). The understanding of the *R*/*Avr* relationship can orient effective LB resistance engineering and breeding (Vleeshouwers and Oliver, 2014; Vleeshouwers *et al*., 2011). More than 20 *R* genes have been isolated from wild species with resistance to LB (Rodewald and Trognitz, 2013). These *R* genes can be introduced simultaneously by genetic engineering into an existing variety without altering any of its properties, except reducing its susceptibility to LB (Haesaert *et al*., 2015; Zhu *et al*., 2012).

In this study, we considered sourcing *R* genes from *S. bulbocastanum* and *S. venturii* because the *RB*,* Rpi‐blb2* and *Rpi‐vnt1.1* genes were reported to confer broad‐spectrum resistance against a wide range of *P. infestans* races (Pel *et al*., 2009; Song *et al*., 2003; Van der Vossen *et al*., 2005). No known isolates were virulent on both wild species and none had been identified that were able to overcome all three *R* genes (Vleeshouwers and Oliver, 2014; Vleeshouwers *et al*., 2011). The *RB* gene, also referred to as *Rpi‐blb1*, has been shown to slow down lesion development and to confer partial field resistance to LB (Chen and Halterman, 2011; Halterman *et al*., 2008; Kuhl *et al*., 2007). A transcript dose–response was documented for resistance to foliar LB, but also for tuber resistance (Bradeen *et al*., 2009; Kramer *et al*., 2009; Millett *et al*., 2015). The *Rpi‐blb2* gene was also shown to confer a high level of field resistance especially when combined with the *RB* gene (Haesaert *et al*., 2015). The *Rpi‐vnt1.1* gene conferred a good level of resistance except towards the senescence period in the field (Jones *et al*., 2014). The cognate avirulence genes were found for all three *R* genes and virulent alleles were identified (Vleeshouwers *et al*., 2011). *Avrblb1* is a member of the effector *ipiO* family of which some variant *ipiO4* can promote virulence directly or indirectly by masking the avirulence effect of other *ipiO* effectors such as *ipiO1* (Champouret *et al*., 2009; Chen and Halterman, 2017a; Chen and Halterman, 2017b; Chen *et al*., 2012; Halterman *et al*., 2010; and Vleeshouwers *et al*., 2008). *Avrblb2* was discovered by an effectoromics approach and one molecular variant was found to be virulent (Oh *et al*., 2009). However, all isolates with the virulent variant had all also the avirulent variants (Oliva *et al*., 2015). *Avrvnt1* was discovered more recently though virulent strains from Poland and the Andean region (Foster *et al*., 2009; Pais *et al*., 2017). In this case, the *Avrvnt1* effector gene is silenced in virulent isolates, which has been recently shown not to be permanent (Román *et al*., 2017; Stefańczyk *et al*., 2017). In an artificial adaptation experiment, neither *RB* nor *Rpi‐blb2* genes selected virulent variants from a single isolate used to inoculate repeatedly or at high doses (Halterman and Middleton, 2012; Orbegozo *et al*., 2016). All these criteria were considered when selecting the *R* genes to stack into susceptible varieties which were selected as farmer and consumer preferred varieties. We have introduced these *R* genes individually and as a stack into four potato varieties, tested their expression in response to *P. infestans* infection, and assessed their resistance to LB in the greenhouse and in the field. Here we report our efforts to develop and test transgenic events from two varieties in the laboratory and in the field for three seasons. Our results show that stacking *R* genes in potato varieties confers complete resistance, likely long‐lasting, in the field.

## Results

### Transgenic potato development

**Figure 1:**
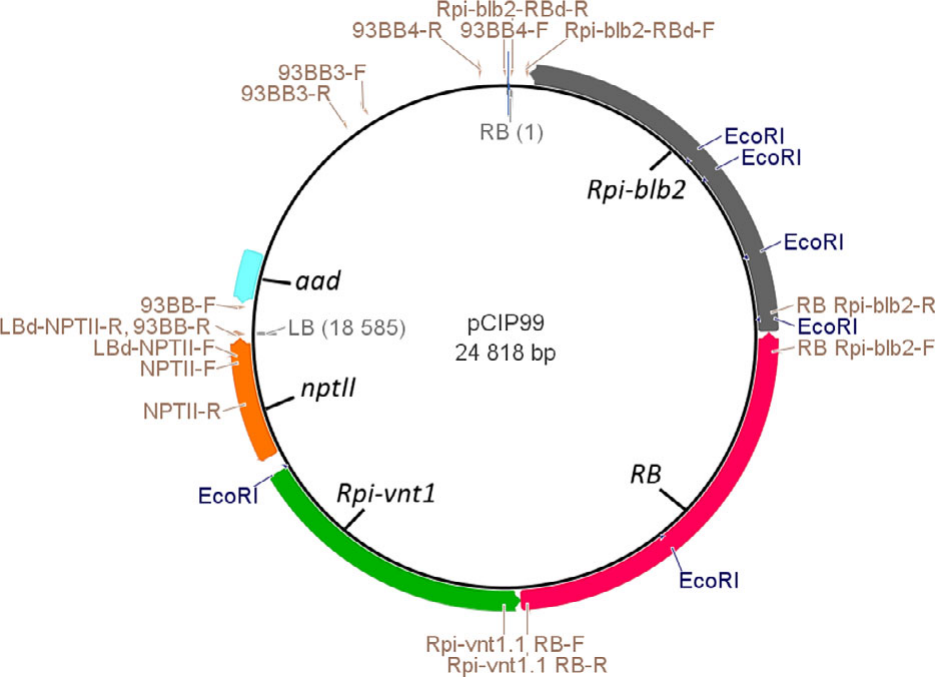
Triple *R* gene stack construct pCIP99 bearing the 18 585 bp T‐DNA from right border RB to left border LB with, clockwise, the three *R* genes (*Rpi‐blb2*,*RB* and *Rpi‐vnt1.1*) and the selectable marker genes (*nptII and aad*) used for transformation of potato and vector selection respectively. Names of the genes and border sequences are indicated inside the circle while primers used to characterize the inserted T‐DNA in the potato plants are indicated outside the circle with their sequences provided in Table S1. Positions of the *Eco* RI sites are also indicated on the outside circle.

**Figure 2:**
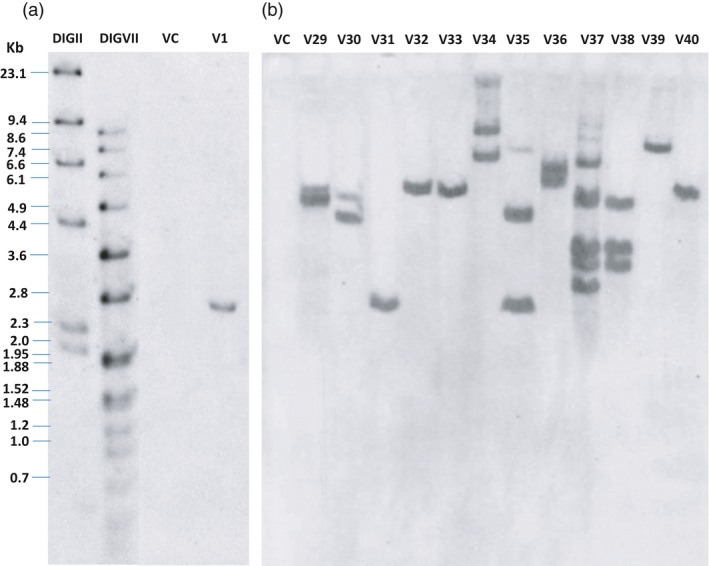
T‐DNA copy number estimated by number of positive bands using southern blotting of transgenic events from ‘Victoria’ with *Eco* RI digested DNA from the 3*R*‐gene stack transgenic events using the *nptII* gene as probe: (a) Single copy of the transgenic event Vic.1 used in field trials. (b) Example of 12 randomly chosen transgenic events bearing the 3R gene stack from ‘Victoria’ variety. VC stands for the non‐transgenic ‘Victoria’ variety, and DIGII and DIGVII are molecular weight standards (Roche) and apply to both gels.

The three *R* genes (*RB*,* Rpi‐blb2* and *Rpi‐vnt1.1*) were assembled in the same transcription direction by cloning unmodified genomic fragments from the wild species: referred to below as the 3*R*‐gene stack (Figure 1). *Agro*‐infection with the vector pCIP99 bearing the 3*R*‐gene stack produced 331 and 149 transgenic events from ‘Desiree’ and ‘Victoria’ respectively. Transformation efficiency of ‘Desiree’ (3.4%) was not affected by the relatively large size of the T‐DNA (18 585 bp). Indeed, transformation efficiency of ‘Desiree’ with single *R* gene construct was previously reported as 3.7% for *RB* gene, 6% for *Rpi‐blb2* gene and 7.5% for *Rpi‐vnt1.1* gene (Orbegozo *et al*., 2016; Román *et al*., 2015, 2017). The variety ‘Victoria’ had lower transformation efficiency (1.5%) and took longer to produce regenerants. We screened transgenic events to identify single copy, absence of vector backbone sequences, and complete T‐DNA insertions which are important if considered in the future for commercial release (Table 1). Southern blotting and PCR had to take into account the presence in the potato genome of numerous homologs of the three *R* genes. Indeed, the analysis of sequence identity between the three *R* genes from the wild species and the potato genome sequence revealed one to three homologs per gene with sequence identity above 88% even for non‐coding regions of the genes (introns, promoters or transcription termination regions). This level of sequence identity would give a large number of positive bands in the background making it difficult to assess the exact number of copies of the new *R* genes. We therefore used *nptII* gene as a probe for southern blotting to assess copy number by counting the number of positive bands for each transgenic event (Figure 2). In addition, the *nptII* gene is next to the left border which enters last into the plant cell, making it a good choice for assessing copy number of the T‐DNA because the rest of the T‐DNA has already entered the plant cell (Horsch and Klee, 1986). However, T‐DNA is sometimes inserted only partially or with partial repeats and therefore additional molecular characterization will be needed to confirm the exact number of copies and its integrity. About 50% of the insertions appeared to be single copy, confirming this parameter used in our study on the cost to produce transgenic events (Schiek *et al*., 2016). PCR amplifications were done between the end and the start of contiguous genes as well as between the T‐DNA border sequence and the first gene to avoid amplifying any of the homologs (Figure 1). We also used three sets of primers to select against the presence of significant portions of the vector backbone (Table S1). Again, here we confirmed that two‐thirds of the transgenic events do not have the vector backbone sequences tested, which is the frequency set by Schiek *et al*. (2016). A total of 26 and 15 transgenic events matched our criteria to be selected as candidate lead transgenic events from ‘Desiree’ and ‘Victoria’ respectively (Table 1).

**Table 1:** Selection of candidate lead transgenic events from ‘Desiree’ and ‘Victoria’ varieties. Screening of the transgenic events proceeded sequentially with absence of vector backbone sequences, presence of the three *R* genes and of the right and left borders, single copy insertions and extreme resistance to late blight by whole‐plant bioassays in confined environment. For ‘Desiree’, we screened for LB resistance before single copy insertion to obtain data of extreme resistance frequency from a larger sample of transgenic events. Numbers in parenthesis represent the number of transgenic events tested. The last row represents the resulting candidate lead transgenic events which matched all five criteria

Variety	Desiree	Victoria
Total number of transgenic events	331	149
Absence of backbone vector sequences	212 (326)	74 (116)
Presence of the 3 *R* genes	194 (194)	86 (94)
Presence of left and right border sequences	nd	71–64 (71)
Single copy of *nptII* gene	27 (44)	24 (46)
Extreme resistance to *Phytophthora infestans* (WPA)	75 (97)	15 (18)
Candidate lead transgenic events	26	15

nd, not determined.

### Combined effect of *R* genes on LB resistance in 3*R*‐gene transgenic events

**Figure 3:**
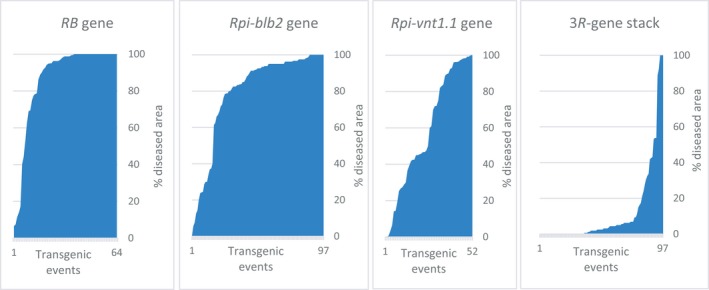
Combined effect of stacking *R* genes to confer extreme resistance to late blight disease. Y axis represents the % of diseased area, whereas the X axis is the transgenic event. From left to right, extreme resistant transgenic events are: two out of 64 transgenic events with *RB* alone, three out of 97 with *Rpi‐blb2*, five out of 52 with *Rpi‐vnt1.1* and 75 out of 97 with the 3*R*‐gene stack.

We observed that the stacking of the three *R* genes has a positive effect on LB resistance as compared to a single *R* gene. This observation comes from the whole‐plant bioassay using the POX067 isolate which is able to infect the ‘Desiree’ and ‘Victoria’ varieties; it was used previously to select transgenic events with one of the three *R* genes, and has been used for selecting the transgenic events with the 3*R*‐gene stack. Indeed, the frequency of extreme resistant transgenic events is rather low when using single *R* genes, as we have reported previously: two out of 64 transgenic events with the *RB* gene alone, three out of 97 with the *Rpi‐blb2* gene, and five out of 52 with the *Rpi‐vnt1.1* gene (Orbegozo *et al*., 2016; Román *et al*., 2015, 2017). However, out of 97 transgenic events we observed 75 with extreme resistance with the 3*R*‐gene stack from the variety ‘Desiree’ (Figure 3). Hence, the frequency of the desirable phenotype (extreme resistance) went from 3% to 9% with single *R* genes to 75% when all three *R* genes were stacked.

### *R* gene expression in the 3*R*‐gene stack transgenic event from ‘Victoria’

All three *R* genes are native genes from the wild species bearing their original, native regulatory promoters, motivating us to verify that all of them were expressed in the 3*R* transgenic events. Thus, we characterized *R* gene expression in the transgenic event with the 3*R*‐gene stack from the variety ‘Victoria’ used in the field trials (Vic.1). This event was chosen because the variety ‘Victoria’ is the most interesting for a first release in SSA countries. The RTqPCR results revealed that all three *R* genes are expressed at a very low level at each time point. The differences between time points and between genes were not significant likely due to the low level of expression (Figure S1). The screening of additional transgenic events from ‘Victoria’ could reveal events with higher *R* gene expression for which pathogen‐induction could be tested.

### LB disease progress in confined field trials (CFTs)

**Figure 4:**
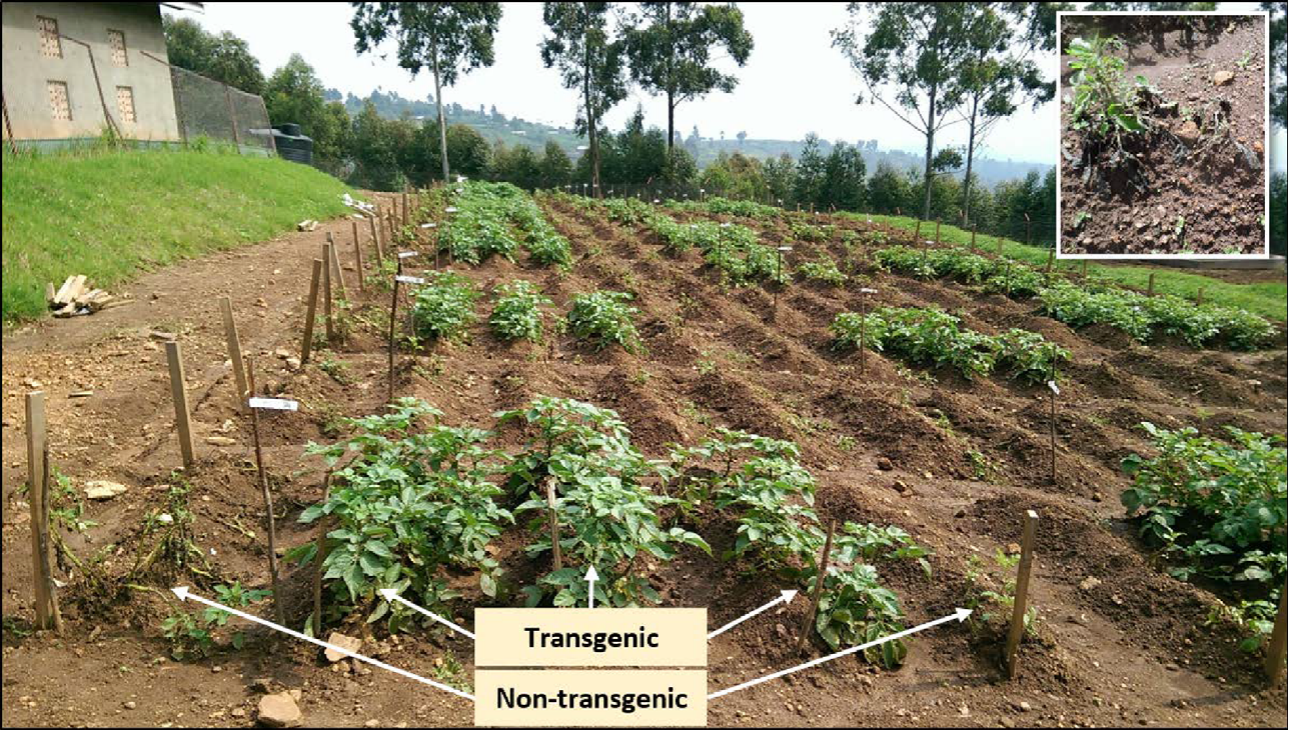
Confined field trial 60 days after planting during the second season (Oct 14, 2015 – Jan 20, 2016) with a close‐up on nontransgenic ‘Victoria’ plant heavily infected by *Phytophthora infestans*. Each plot is made of three rows of transgenic plants flanked by one row of spreaders on each side as indicated for the front plot. Green potato rows are all transgenic events and ‘Cruza 148’, whereas the empty rows are the non‐transgenic rows of ‘Desiree’ and ‘Victoria’.

**Figure 5:**
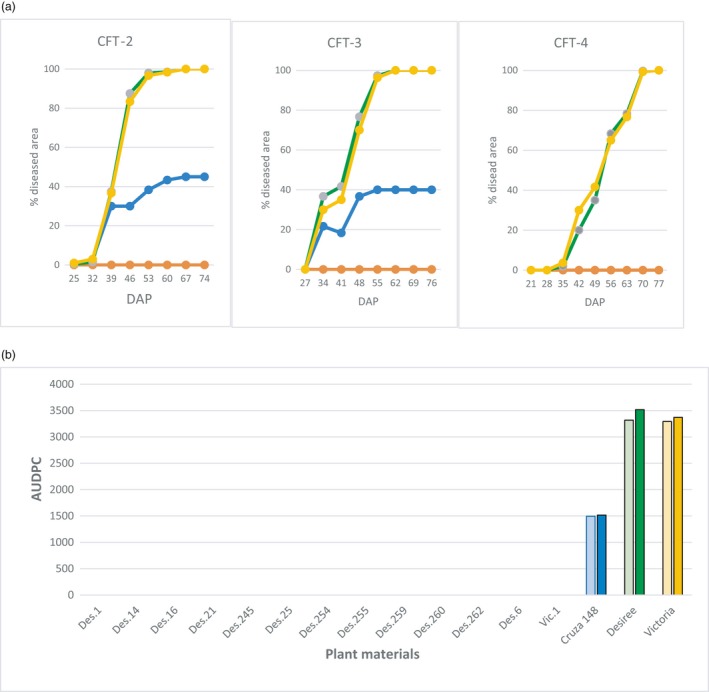
Late blight disease in confined field trials. (a) Disease progress curve during three seasons (CFT‐2, CFT‐3 for 12 transgenic events from ‘Desiree’ and one from ‘Victoria’, and CFT‐4 for two transgenic events Des.255 and Vic.1 under natural infestation. Y and X axes are % foliage affected and days‐after‐planting respectively. Green line is ‘Desiree’, yellow is ‘Victoria’, and blue is ‘Cruza 148’, whereas the orange line is all 13 transgenic events. (b) Area under the disease progress curve (AUDPC) calculated from the estimated percentages of foliage affected recorded during two seasons (CFT‐2 pale colour bar next to CFT‐3 with plain colour).

The potato plants in the field were exposed to natural infection by *Phytophthora infestans* using a randomized complete block design of three repetitions (Figure S2). The transgenic events planted were all resistant in the whole‐plant bioassays, but with variable degree of damaged areas (Table S2). The development of LB on leaves was monitored weekly 3 weeks after emergence during CFT‐2, CFT‐3 and CFT‐4. The disease usually started 30–45 days after planting and was observed only on non‐transgenic materials in all three CFTs. ‘Cruza 148’, a moderately resistant variety, reached 40–45% affected foliage, whereas ‘Desiree’ and ‘Victoria’ were all dead 20 days after the disease had started. None of the transgenic events presented lesions caused by *P. infestans* at any time (Figure 4). Even at foliage senescence when plant defences are decreasing, no lesions were observed on the transgenic plants, indicating that the tubers would not be infected by the pathogen and therefore would not spread it after replanting. The AUDPC value for CFT‐2 and CFT‐3 were close to 1500 for ‘Cruza 148’ and above 3000 for ‘Desiree’ and ‘Victoria’ while it was null for all transgenic events (Figure 5). CFT‐4 was done with one transgenic event per variety to assess whether the *R*‐gene mediated resistance had any effect on yield. Des.255 was chosen arbitrarily among the 12 transgenic events and Vic.1 was the only one available at that time.

### *Phytophthora infestans* population and *Avr* gene expression

Pathogen isolates from the field site were characterized using infected leaves of ‘Desiree’ and ‘Victoria’ non‐transgenic plants between 2014 and 2016. A changeover of the *P. infestans* clonal lineage was noted during this time period. Samples collected in 2014 from the mock trial (*n* = 12) revealed *P. infestans* belonged to the ‘old’ US‐1 lineage; then in 2015, during the first season there were 42 samples of 2_A1 (80%) from CFT‐1 (*n* = 52) and in the second season of 2015 there were 24 samples of 2_A1 (90%) from CFT‐2 (*n* = 27; Table S3). Then, all the samples in 2016 from CFT‐3 (*n* = 14) and CFT‐4 (*n* = 25) were of the 2_A1 lineage which originally had been described as KE‐1 before its European origin was demonstrated (Njoroge *et al*., 2016). We analysed the presence and expression of the *Avr* genes corresponding to the three *R* genes in both lineages to investigate the durability of the disease resistance of the 3*R*‐gene transgenic potatoes. The US‐1 isolates collected during the mock trial and subsequent CFT seasons expressed neither the avirulent nor the virulent effector variants of *Avrblb1*. However, the same isolates expressed all four *Avrblb2* effector variants as well as the *Avrvnt1* effector gene. The 2_A1 isolates expressed only the avirulent *Avrblb1* allele variants (*ipiO1* and *ipiO2*). For the *Avrblb2* effector gene, two effector‐genotypes within the 2_A1 lineage were detected; one expressing the four *Avrblb2* allele variants and another one which did not express the virulent variant allele, *Phe69*. All 2_A1 isolates expressed the *Avrvnt1* effector gene. Hence, all isolate samples collected in the last 2 years indicate complete displacement of US‐1 and confirmed that the *P. infestans* strain present at the CFT in southwestern Uganda belongs to the new aggressive strain 2_A1 which expresses all cognate avirulence effector genes.

### Tuber morphology and yield assessment without fungicide protection

**Figure 6:**
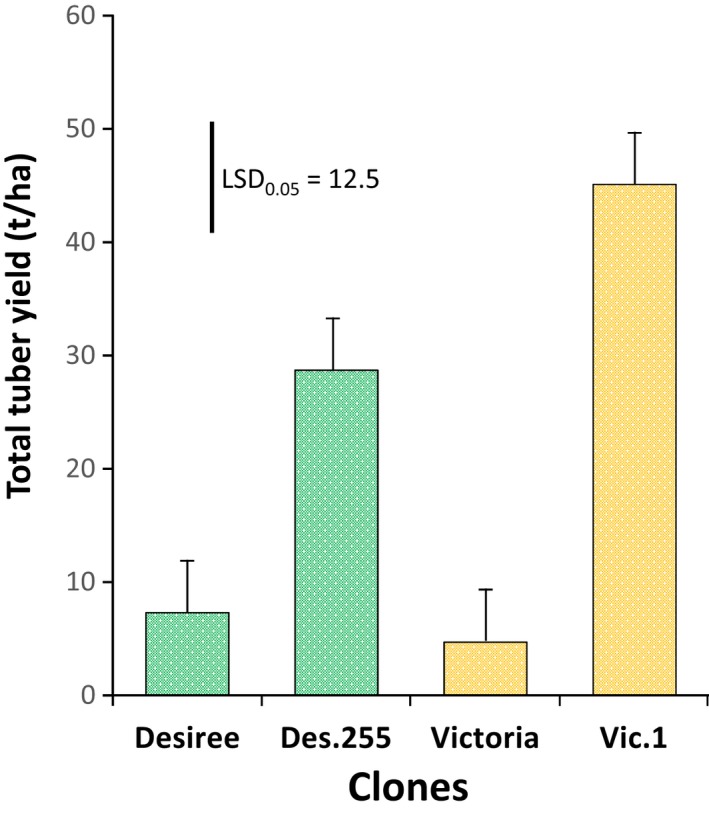
Total yield of non‐transgenic and transgenic ‘Desiree’ and ‘Victoria’ at KaZARDI, southwest Uganda in the second cropping season of 2016 (CFT‐4). Des 255 and Vic.1 are the transgenic events from ‘Desiree’ and ‘Victoria’ respectively. The error bar is measured and nipped from the *y*‐axis. Value of error bar determined from lsd test at 0.05 probability is 12.47.

Harvest of the potato trials took place at 97 days after planting (dap) for CFT‐2 and 105 dap for CFT‐3. The physiological and maturity conditions of the planting material (seed tubers) were too variable to assess yield. Indeed, the variations between blocks for the same genotype were higher than between events of the same variety. However, we characterized the tubers morphologically in order to identify any potential off‐type transgenic event. Little variation was observed for skin and flesh colour, or for shape (Table S4). The differences that did occur were within the natural variation expected in a field harvest. We performed a new CFT for estimating yield using better quality tuber seeds and one transgenic event from each variety: Des.255 was chosen randomly from the 12 field‐tested transgenic events from ‘Desiree’, and Vic.1 was chosen because it was the only transgenic event available at that time from ‘Victoria’. The yield of the transgenic events and non‐transgenic varieties was estimated from unsprayed plots after harvest of the CFT‐4 (110 dap). This was done to reflect the situation of small‐scale farm holders who do not spray until disease lesions appear on the crop. Des.255 and Vic.1 presented total yields at 29 and 45 t/ha, respectively, whereas their unsprayed non‐transgenic equivalents did not produce marketable tubers at all (Figure 6).

## Discussion

LB affects potato and other Solanaceae crops worldwide, and has been named ‘the infamous disease’ because of the massive destruction of the potato crop which contributed to the deaths of millions of Irish and other European people during the middle of the 19th century. Our understanding of the interactions between *Phytophthora infestans* and the potato, the availability of genome sequences from both organisms and of new tools to genetically improve potato varieties allow us to progress towards an effective control of this devastating disease. The research reported here is a first proof that such an objective is no longer simply idealistic.

We selected resistance genes of the effector‐triggered‐immunity (ETI) system, which is most effective during the biotrophic phase of *P. infestans,* for their known broad‐spectrum resistance and high efficacy without yield penalty. Two genes (*RB*, and *Rpi‐blb2*) from *Solanum bulbocastanum* and one gene (*Rpi‐vnt1.1*) from *S. venturii* were stacked into farmers’ preferred varieties grown in sub‐Saharan Africa. Single *R* gene and 3*R‐*gene stack transgenic events have been produced from the variety ‘Desiree’. The combined effect of the three *R* genes on resistance to LB was notorious; about 75% of the transgenic events with the 3*R*‐gene stack were found with extreme resistance, whereas only 3%–5% of events with single *R* genes expressed extreme resistance. This is greater than expected by simple additive effects and explains previous observations of observed high frequencies of resistant transgenic events using *R* gene stacks (Haesaert *et al*., 2015; Zhu *et al*., 2012).

Early on, *R* gene expression was shown to be induced one or 2 days after inoculation with *P. infestans,* slowly decreasing over the next 3 days (Kramer *et al*., 2009; Orbegozo *et al*., 2016; Román *et al*., 2017; Vleeshouwers *et al*., 2011). We observed *R* gene expression in the 3*R*‐gene stack transgenic event, but at such a low level that statistically significant differences could not be observed. Nevertheless, high level resistance was observed in the whole‐plant assays.

The pathogen diversity in SSA is remarkably low. *P. infestans* was sampled from lesions on susceptible potato plants from the confined field trials for 3 years. In the first year, most isolates belonged to the ‘old’ US‐1 lineage with the presence of the 2_A1 lineage already detected in 2007 in a few fields in Kenya and referred to as KE‐1 at that time (Njoroge *et al*., 2016). The 2016 samples revealed, however, that all of them pertained to the same lineage, 2_A1, and expressed the cognate *Avr* effector genes of the 3*R*‐gene stack. Hence, the absence of the A2 mating type, the dominance of the 2_A1 lineage with low genetic diversity, and the expression of all cognate effector genes are excellent premises for the extreme resistance to LB disease of the 3*R*‐gene transgenic events to be long‐lasting. However, the pathogen population will need to be monitored seasonally, at least in the regions where the 3*R*‐gene potato will be released, for the presence of US‐1 on potato and the emergence of new strains which seems inevitable due to increasing seed potato trade with Europe.

All 13 transgenic events bearing the 3*R*‐gene stack, characterized as extremely to moderately resistant using a whole‐plant bioassay in the greenhouse, were all completely resistant in all field trials. Not a single leaf was found with a *P. infestans* lesion, unlike the greenhouse observations and unlike field observations with single *R* genes on senescent leaves (Haesaert *et al*., 2015; Jones *et al*., 2014). In the whole‐plant bioassay in the greenhouse, the pathogen is at high inoculum density with saturated humidity which may cause damaged leaves resulting from hypersensitive responses occurring over a large area saturated by the pathogen. In agricultural fields, the inoculum is found at much lower levels, which explains why no hypersensitive reaction damage was observed in the field. The third field trial with one transgenic event from ‘Desiree’ and ‘Victoria’ resulted not only in the third confirmation of the extreme resistance, but also a first estimation of their yield under no‐fungicide control conditions. We chose to estimate yield in the absence of control by fungicides because it is the yield small‐farm holders should expect to obtain since they only spray when the see clear disease lesions on the leaves of the crop. Yield and morphological characteristics of the tubers were not significantly different from those obtained from the non‐transgenic varieties protected by fungicides. This coincides with other reports showing that *R*‐gene‐mediated resistance does not affect yield or tuber characteristics (Halterman *et al*., 2008; Jones *et al*., 2014). In the future, yield will be estimated under fungicide spray condition for both transgenic and non‐transgenic potato to verify the 3*R*‐gene stack does not confer yield penalty.

In conclusion, this study has demonstrated that potato transgenic events bearing the 3*R*‐gene stack have acquired complete resistance to LB disease based on field trials during three consecutive seasons in southwestern Uganda. The absence of lesions during senescence also indicates that seed quality will not be affected by the pathogen. The observed marketable and total yield for the transgenic events appeared to be similar to the yield of their non‐transgenic counterpart produced by small‐scale farm holders under similar conditions but protected by fungicides. The 3*R*‐gene potato yields were three to four times above the national average. In addition, the single mating type and low diversity of the pathogen expressing cognate effector genes of the three *R* genes used in the stack augur that the resistance will be durable. The next steps will include the identification of the lead transgenic event which will be commercialized. This will be achieved after characterizing the insertion into the potato genome, by verifying that the *R* genes are functional, there are no vector backbone sequences, and that no long ORF (open reading frames) are produced at the junction of the T‐DNA and the potato flanking sequences. In addition, multi‐locational trials for two or three seasons will be needed to develop regulatory studies to ascertain that the transgenic potato does not pose risks to human or animal health, or to the environment. The commercialization of the transgenic potato is not immediately possible in Uganda until the recently revised biosafety bill is approved by the parliament and the president. The absence of a legislation for commercialization of transgenic crops is common to most sub‐Saharan African countries except for Burkina Faso, Sudan and South Africa. Finally, the long‐term sustainability of *R*‐gene‐mediated resistance against LB will be achieved by developing new transgenic events with new *R*‐gene stacks which will be deployed when *P. infestans* eventually overcomes the released 3*R*‐gene variety.

## Experimental procedures

### Plant transformation vector and bacterial strains

The *RB* gene was provided by Prof. J. Jiang's lab at University of Wisconsin with flanking *Bam*HI restriction sites (Song *et al*., 2003). The *Rpi‐vnt1.1* gene of 4310 bp (GenBank accession FJ423044.1) was chemically synthesized with flanking *Xma*I restriction sites and subcloned into the vector pUC57 by GenScript Corp. (Piscataway, New Jersey). The *Xma*I fragment containing *Rpi‐vnt1.1* was subcloned into a *Xma*I‐restricted and dephosphorylated pCAMBIA2300 plant transformation binary vector, creating gene construct pCIP93. The selectable marker gene for vector selection in bacteria is the aminoglycoside phosphotransferase gene conferring resistance to kanamycin. The *Rpi‐blb2* gene was chemically synthesized by Entelechon GmbH (Germany) using nucleotides 1–5730 of the 7967 bp genomic fragment bearing the full *Rpi‐blb2* gene (Genbank accession number DQ122125.1), with flanking *Sbf*I restriction sites, and subcloned into the pEN08E vector. Then the *Sbf*I fragment containing the *Rpi‐blb2* gene was subcloned into a *Sbf*I‐restricted dephosphorylated pCIP93, creating pCIP96. Finally, the *Bam*HI fragment containing the *RB* gene was subcloned into a *Bam*HI restricted and dephosphorylated pCIP96. The resulting gene construct, referred to as pCIP99, bears from the right border the *Rpi‐blb2*,* RB* and *Rpi‐vnt1.1* gene and the *nptII* gene for kanamycin resistance near the left T‐DNA border (Figure 1). pCIP99 was transferred by electroporation into the *Agrobacterium tumefaciens* hypervirulent strain EHA105 (Hood *et al*.,^1993^). R gene homology analyses were done using the genome sequence of a potato from the *S. tuberosum* group Phureja DM1‐3 516 R44 with BLASTn at http://solanaceae.plantbiology.msu.edu/pgsc_download.shtml.

## Plant materials

‘Desiree’ and ‘Victoria’ used for genetic transformation were obtained from the gene bank of the International Potato Center (CIP) as the accession CIP800048 and CIP381381.20 respectively. Both accessions were genotyped by SSR markers to ensure identity with varieties grown under same name in Africa. ‘Desiree’ is a variety that is grown in Kenya which is very susceptible to LB, but has good qualities for table potato. It has oval to round tubers with red skin and cream flesh and a long tuber dormancy. The crop is late maturing (4–4.8 months) and has low to medium yield (30–40 t/ha). ‘Victoria’ (known as ‘Asante’ in Kenya) is a potato variety which was officially released in Uganda in 1992. It has large round tubers with pink smooth skin and pale‐yellow flesh, and a short dormancy (1–1.5 month). The crop has medium maturity (3 months) and has low to medium yield (35–45 t/ha).

### Potato genetic transformation, transgenic event selection and LB resistance assays

*Agrobacterium*‐mediated transformation, the identification of transgenic events by molecular techniques, and their LB resistance assessment were achieved using protocols which have been described in the single gene transformation studies reported previously (Orbegozo *et al*., 2016; Román *et al*., 2017). The PCR molecular characterization of the 3*R*‐gene stack considered sequences with no homologs in the potato genome: (1) the overlapping regions between two consecutive *R* genes; (2) the region between border sequences and the end of the T‐DNA; and (3) the vector backbone sequences. Positions of primers are indicated on Figure 1 and their sequences and amplification conditions are given in Table S1. Primers for making the Southern blotting probe from *nptII* gene are also included. Amplification conditions were standard: 1 denaturation step of 4 min at 94 °C; 35 cycles of 20 s at 94 °C + 20 s at annealing temperature + 1 min at 72 °C; and one terminal elongation step of 5 min at 72 °C. Transformation efficiency (%) was defined as the number of PCR‐positive plants divided by the number of infected explants.

Whole‐plant bioassays of transgenic events were assessed for resistance at 45 days after planting using plants originating from tubers after one generation out of *in vitro* conditions. Assays were performed under controlled conditions in the biosafety greenhouse with a regime of 14 h light at 14–21 °C and 10 h dark at 12–15 °C. Relative humidity was held at 80%–100% throughout the assessment period. The inoculation was done on whole plants by spraying a suspension of 3000 sporangia/mL of the aggressive *P. infestans* isolate POX067 which was collected in Peru in the department of Pasco, zone Oxapampa, and belongs to the EC‐1 lineage. Extreme resistance was defined as less than 10% of leaf damage without sporulation. We used transgenic events with high level resistance Desiree[*Rpi‐blb2*]30, Desiree[*RB*]70, Desiree[*Rpi‐vnt1.1*]55 as transgenic resistance controls, non‐transgenic ‘Desiree’ and non‐transgenic ‘Victoria’ both highly susceptible, and the LBr40 breeding line with high level of resistance.

### *R* gene expression in the triple *R* transgenic event

Transcript level of the *RB*,* Rpi‐blb2 and Rpi‐vnt1.1* genes in *P. infestans* – infected plants was quantified by RT‐qPCR in the transgenic event from Victoria which was field trialled (Vic.1). The analysis was done 1 day before inoculation, and one, three, and 5 days after inoculation. RNA was extracted using the RNeasy Plant Mini Kit (Qiagen, Venlo, The Netherlands) using leaves frozen with liquid nitrogen from greenhouse‐grown plants. Three repetitions of samples and RNA extractions (biological replicates) per treatment were performed. RNA concentration was estimated by spectrophotometry using a NanoDrop Micro Photometer (Thermo Scientific, Waltham, MA) and its integrity with Agilent RNA 6000 Nano Kit. It wasthen converted to cDNA using the Superscript III First‐Strand kit synthesis system for RT‐PCR (Invitrogen, Carlsbad, CA) and Oligo‐dT following the manufacturer′s protocol. Real‐time quantitative PCR (RT‐qPCR) reactions were performed in an optical 96‐well plate with a StepOne Real‐Time System using BrightGreen 5X qPCR Master Mix‐ROX (Applied Biosystems, Foster City, CA). Reactions were prepared in a total volume of 10 μL as follows: 6.4 μL of nuclease‐free water, 2 μL of BrightGreen 5X qPCR Master Mix‐ROX, 1 μL of template cDNA (50 ng) and 0.3 μL (300 nm) of each gene‐specific primer. RT‐qPCR experiments were as follows: 10 min at 95 °C, 40 cycles of 15 s at 95 °C, 1 min at the annealing temperature (55 °C for the *RB*,* Rpi‐blb2* and Ef‐1α transcripts; 57°C for the 57 alpha tubulin transcripts; and 58 °C for the *Rpi‐vnt1.1* transcripts), and 1 min at 60 °C. Amplicon dissociation curves, that is melting curve, were recorded after cycle 40 by heating from 60 to 95 °C with a ramp speed of 0.5 °C/min. We used the average of two reference genes to normalize the abundance of the transcripts of the 3 *R* genes as recommended to study small expression differences (Vandesompele *et al*., 2002). The elongation factor 1 alpha primer (Ef‐1α) previously identified as the best candidate as a reference gene when performing RT‐qPCR with RNA from potato inoculated with *P. infestans* (Nicot *et al*., 2009; Orłowska *et al*., 2012). Second, the 57 alpha‐tubulin (tub) reported as suitable reference gene for the gene expression analysis of *RB* transcripts (Kramer *et al*., 2009). The relative expression values of the *RB*,* Rpi‐blb2* and *Rpi‐vnt1.1* gene were analysed using primers qRT‐RB‐F 5′CAC GAG TGC CCT TTT CTG AC‐3′ and qRT‐RB‐R 5′ACA ATT GAA TTT TTA GAC TT‐3′ (Kramer *et al*., 2009); qRT‐Rpi‐blb2‐F 5′TTC AAA ACC CCA AAT AAG TTT CAA C‐3′ and qRT‐Rpi‐blb2‐R 5′CCA TGC TTG CTG TAC TTT GCA‐3′ (EPA Ireland, 2006); qRT‐Rpi‐vnt1.1‐F 5′GGT AAG GTA TTG GCT CTG‐3′ and qRT‐Rpi‐vnt1.1‐R 5′CTT CTC AGC AAT CCA CAT A‐3′ (Román *et al*., 2017). CT values were analysed using Relative Expression Software Tool (Pfaffl *et al*., 2002; http://rest.gene-quantification.info/). A two‐way ANOVA followed by Dunnett multiple comparison test was used to establish significant differences in relative expression of the *RB*,* Rpi‐blb2 Rpi‐vnt1.1* gene between the transgenic events at pre‐inoculation stage. The statistical analyses were performed using GraphPad Prism (GraphPad Software, Inc., La Jolla, CA).

### *P. infestans* population and *Avr* effector gene expression analyses

The *P. infetsans* samples used in this study were collected from LB infected leaflets with single sporulating lesions on Whatman FTA card (GE Healthcare UK, Buckinghamshire, UK) and in RNA*later* solution (Qiagen). The FTA card samples were used for genotyping by a single 12‐plex microsatellite protocol (Njoroge *et al*., 2016). The RNA*later* samples were used for effector allele expression and for genotyping with two SSR markers which differentiate the prevailing clonal genotypes of *P. infestans* in east Africa. A total of 34 samples on FTA cards and 96 samples in RNA*later* sampled during the mock trial and the four confined field trials were analysed. Total RNA was extracted using the RNeasy Plant Mini Kit (Qiagen) and the cDNA synthesized using the RevertAid First Strand cDNA Synthesis Kit (Thermo Fisher Scientific) following the protocols provided by the manufacturers. Multiplex SSR genotyping was performed with primer concentrations between 0.16 μm and 0.3 μm as described by Li *et al*. (2013). Reactions were carried out in a 12.5 μL volume consisting of 6.25 μL of 2X Type‐it Master Mix (Qiagen), 1.25 μL of a 10× multiplex primer mix, 2 μL of nuclease‐free water and 3 μL of the FTA eluent. Amplification conditions were as follows: 5 min at 95 °C, followed by 33 cycles of 30 s at 95 °C, 90 s at 58 °C, 30 s at 72 °C, and a final extension at 60 °C for 30 min. The single primer reactions used AccuPower^®^ Taq PCR 2X Master Mix (Bioneer, Daedeok‐gu, Republic of Korea), 2 μL of template cDNA and primers at a final concentration of 0.25 μm. Amplification conditions were as follows: 3 min at 94°C; followed by 33 cycles of 30 s at 94°C, 30 s at 62°C (Pi04, PiG11), 60 °C (Pi16), 58 °C (Pi56, Pi70, Pi89, Pi4B) or 50 °C (D13), 45 s at 72 °C for and completed with a final extension at 72 °C for 25 min. Allele‐specific primers were used to detect the *Avrblb1* allele variants (*ipiO1*,* ipiO2*,* ipiO3* and *ipiO4*), *Avrblb2* allele variants (*Avrblb2* ^*Ala69*^, *Avrblb2* ^*Ile69*^ *, Avrblb2* ^*Phe69*^ and *Avrblb2* ^*Val69*^) and the *Avrvnt1* effector gene. The cDNA was first screened via PCR for the presence of *Avr3a* effector gene, the indicator for the biotrophic phase of *P. infestans*. All reactions were carried out in a total reaction volume of 10 μL containing 5 μL of AccuPower^®^ Taq PCR 2x Master Mix (Bioneer), 0.5 μL of each primer pair (5 μm), 2 μL of cDNA template and 2 μL of nuclease‐free water. Amplification conditions were as follows: 3 min at 94 °C; followed by 32 cycles of 30 s at 94 °C, 30 s at annealing temperature, 72 °C for 45 s, and a final extension at 72 °C for 5 min. Primer sequences, annealing temperature and amplicon sizes are provided in Table S1. These were obtained as follows: *Avrblb1* variants – R. Oliva (pers. comm.); *Avrblb2* variants from Oliva *et al*. (2015); and *Avrvnt1* and *Avr3a* from Pel (2010). For the detection of effector alleles *Avr3a*,* ipiO*4, *Avrblb2* ^*Phe69*^and *Avrvnt1*, a touchdown step in the PCR reaction was included.

### Confined field trials

We tested the 3*R*‐gene potatoes in the field in the heart of the potato cultivation region in southwestern Uganda where LB damages the potato crop every year. Considering molecular criteria and greenhouse whole‐plant bioassay, we selected 12 transgenic events from ‘Desiree’ and one from the variety ‘Victoria’, which was the only one available at that time. We distinguished three classes of leaf damages (none, low and moderate), although when the damaged areas were observed, they were without sporulation (Table S2). A confined field trial site was constructed within the premises of the Kachwekano Zonal Agricultural Research and Development Institute (KaZARDI) station near Kabale in southwestern Uganda (geographic position 1°15′19.0″S 29°56′34.3″E). It complied with the requirements exposed in the ‘National Guidelines for Containment: For Regulation of Research with Genetically Modified Organisms and Microbes’, May 2007. ‘Decision Document’ and ‘Research Approval’ permits were granted by Ugandan competent authority (NBC Decision Number 1/2015 on May 6, 2015; UNCST Research Approval Number A490 on June 12, 2015).

### CFT planting and field design

Sprouted seed tubers were obtained from greenhouse potted plants for the first trial CFT‐1, whereas for the next three seasons (CFT‐2, CFT‐3 and CFT‐4), seed tubers used were obtained from the previous season harvest following seed quality checks. A randomized complete block design (RCBD) with three replications was used for all four CFT conducted between 2014 and 2016 (Figure S1). A total of 48 experimental plots were used during CFT‐1, ‐2 and ‐3. For each replication, the 16 plots were planted as follows: 12 transgenic events from ‘Desiree’, one transgenic event from ‘Victoria’, ‘Desiree’ ‘Victoria’, and ‘Cruza 148’. Each plot, separated from each other by 1 m, consisted of five rows at spacing of 75 cm. Five seed tubers were planted at spacing of 30 cm within rows. The two border rows of the five rows plots were planted with non‐transgenic ‘Victoria’ to spread the disease, whereas the three central ones were planted with the material under testing. For CFT‐4, we used the same distance between rows, no spreader rows as found unnecessary, but used 12 plots of three rows planted with 10 seed tubers of the material under testing which were as follows: one ‘Desiree’ transgenic event (Des.255), one ‘Victoria’ transgenic event (Vic.1), ‘Desiree’ and ‘Victoria’. At planting, 300 kg/ha of NPK 17:17:17 fertilizer was applied. Weeding was done five times while control for cutworms, aphids and leaf‐miners was done using systemic insecticides. Flower buds were removed every 2 days during the flowering period in compliance with biosafety regulation. Volunteer potato plants were monitored both within the field and within 100 m radius around the CFT site during the conduct of all trials. The field trials were left under natural infection by the LB pathogen without using any fungicide sprays.

### LB disease progress in the CFT

The development of LB on leaves was monitored on a weekly basis. Disease severity and plant leaf area affected (PLAA) were scored following Forbes *et al*. (2014). Data were logged in a field data sheet in a handheld mobile computer (Motorola MC9500K). Re‐growth after the disease had damaged plants was not accounted for. The amount of disease was computed as area under disease progress curve (AUDPC) using procedure of Campbell and Madden (1990):

$$\text{AUDPC}=\sum_{i=1}^{n}\left[\left(X_{i}+X_{i-1}\right)/2\right]\left[t_{i}-t_{i-1}\right]$$

where: *X*_*i*_ = present disease severity, *X*_*i*−1_ = previous disease severity, *t*_*i*_ − *t*_*i*−1_ = time difference between two consecutive disease severity measurements, *n* being the total of number of measurements.

The KaZARDI station where the confined field trials took place has high disease pressure for LB due to its high altitude (2200 masl), high humidity and low temperatures 14–25 °C during the two rainy seasons (October – January, and March – June). These conditions favour the spread of *Phytophthora infestans* and therefore plants were exposed to natural infection.

### Harvest, off‐type and yield assessment

The procedures used are those practiced for harvest of conventional potato clones. Briefly, the aboveground parts of the plants are cut off (dehaulming) and placed in the incineration pit located within the CFT. Using grub hoes, the tubers were dug out and kept all together for each plot. Tubers are classified as follows: category 1 = small tubers > 30 mm, category 2 = 30–45 mm, category 3 = 30–60 mm. Each category is numbered and weighed. One tuber of the category 3 is cut transversally to appreciate flesh colour. Tuber shape and skin colour were observed visually. Yield assessment was conducted using the data for category 2 and 3 (marketable potatoes) obtained from CFT‐4. Total tuber yield in kilograms per plot was standardized to hectare basis after correcting for missing hills by dividing the weight of harvested tubers by 6.75 and multiplying the quotient by 10 to obtain yield in tons per hectare (t/ha). Being a single treatment factor, with four levels, in randomized complete blocks experiment, the significance of treatments was tested using one‐way analysis of variance with blocking model in GenStat Release 11.1 software. Means for levels of the significant factors were compared using Fishers’ Protect Least Significant Difference test at 5% probability. Pertinent graphs were generated using MS Excel to elucidate variations in treatment factor levels.

## Competing financial interests

The authors declare no competing financial interests.

## Authors 'contributions

M.G. conceived and oversaw the research from its start and wrote the manuscript with input from all other authors. J.C.T. developed the pCIP99 gene construct in Peru. C.R. and M.L.R. produced the transgenic events in Peru from ‘Desiree’ and ‘Victoria’ respectively. C.R. also generated the gene expression studies with J.K. and coordinated the pathogen resistance bioassays with S.G. in the greenhouse, all in Peru. E.M. generated seed tubers in Kenya, obtained permits, and participated in field trials in Uganda. A.A.B., A.B., and A.K. developed confined field trials in Uganda including facility construction, obtaining permits, and generated the disease and yield data. A.N. assisted in trial construction, permits, and did the pathogen sampling and *Avr* gene expression analyses both in Uganda and Kenya. G.F. oversaw the pathology work in the laboratory and in the greenhouse in Peru. Finally, A.K. managed the Ugandan research and all administrative aspects.

## Supporting information


**Figure S1** Gene expression of the *RB*,* Rpi‐blb2* and *Rpi‐vnt1.1* gene in the triple *R* gene transgenic event Vic.1 from the variety ‘Victoria’ relative to the expression 1 day before infection (dbi) by *P. infestans* strain POX067.
**Figure S2** Field layout of the confined field trials.
**Table S1** List of primers and amplification conditions used to characterize T‐DNA insertions into potato varieties (presence of the *R* genes and *nptII* gene, completeness of T‐DNA towards both ends, and absence of vector backbone sequences – see Figure 1) and the cognate effector genes in *Phytophthora infestans* field samples (*Avr3a* is an internal reference gene).
**Table S2** Transgenic materials selected for field evaluation based on average of leaf damage in whole‐plant bioassays in greenhouse resistance tests.
**Table S3** Expression of the *Phytophthora infestans* effector genes from isolates collected on late blight infected non‐transgenic potato control plants during the mock trial and the subsequent confined field trials.
**Table S4** Morphology of tubers harvested from transgenic events and their control varieties from two seasons (CFT‐2 and CFT‐3).
