# Supplementary material for: Stacking three late blight resistance genes from wild species directly into African highland potato varieties confers complete field resistance to local blight races
Source: Plant Biotechnol J. 2018 Dec 21;17(6):1119–29. doi: 10.1111/pbi.13042 (PMC6523587; doi:10.1111/pbi.13042)
Supplement: Supplementary file 1 — Figure S1 Gene expression of the RB, Rpi‐blb2 and Rpi‐vnt1.1 gene in the triple R gene transgenic event Vic.1 from the variety ‘Victoria’ relative to the expression 1 day before infection (dbi) by P. infestans strain POX067. Figure S2 Field layout of the confined field trials. Table S1 List of primers and amplification conditions used to characterize T‐DNA insertions into potato varieties (presence of the R genes and nptII gene, completeness of T‐DNA towards both ends, and absence of vector backbone sequences – see Figure 1) and the cognate effector genes in Phytophthora infestans field samples (Avr3a is an internal reference gene). Table S2 Transgenic materials selected for field evaluation based on average of leaf damage in whole‐plant bioassays in greenhouse resistance tests. Table S3 Expression of the Phytophthora infestans effector genes from isolates collected on late blight infected non‐transgenic potato control plants during the mock trial and the subsequent confined field trials. Table S4 Morphology of tubers harvested from transgenic events and their control varieties from two seasons (CFT‐2 and CFT‐3). [file PBI-17-1119-s001.docx]

**Supplementary information:**


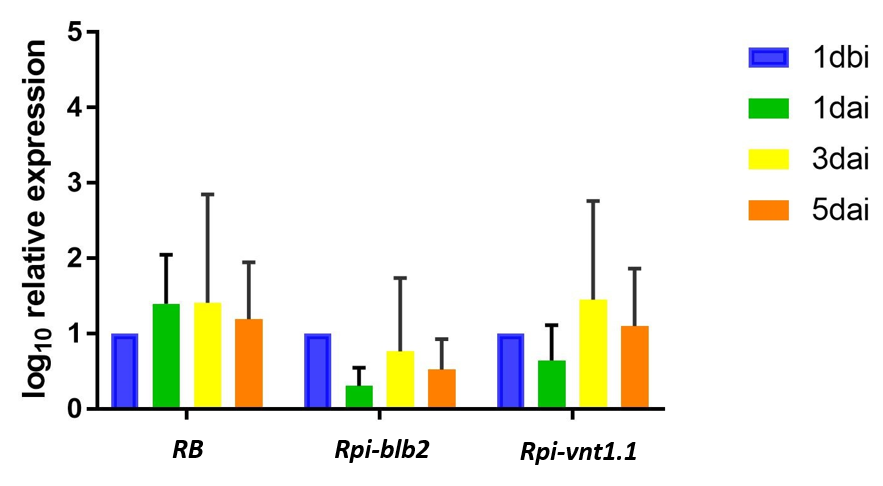


**Figure S1**: Gene expression of the *RB*, *Rpi-blb2* and *Rpi-vnt1.1* gene in the triple *R* gene transgenic event Vic.1 from the variety ‘Victoria’ relative to the expression one day before infection (dbi) by *P. infestans* strain POX067. Three time-points were 1 dai (first day after infection), 3 dai, and 5 dai. Bars represent the mean of three biological replicates and three technical replicates with their corresponding standard deviation.

**A**

**B**

**Figure S2**: Field layout of the confined field trials. A. CFT-2 and 3 were the same randomly-chosen block design (three blocks or repetitions) of 12 transgenic events from ‘Desiree’ (Des.255), one from ‘Victoria’ (Vic.1), the non-transgenic varieties ‘Desiree’, ‘Victoria’ and ‘Cruza’ (for Cruza 148), and spreader rows with ‘Victoria. B. CFT-4 was randomly-chosen block design (threee blocks or repetitions) of Des.255, Vic.1, and the non-transgenic varieties ‘Desiree, ‘Victoria’.

**Table S1**: List of primers and amplification conditions used to characterize T-DNA insertions into potato varieties (presence of the *R* genes and *nptII* gene, completeness of T-DNA towards both ends, and absence of vector backbone sequences – see Figure 1) and the cognate effector genes in *Phytophthora* *infestans* field samples (*Avr3a* is an internal reference gene). A. t. stands for annealing temperature.

| Target region  Primer name | Forward primer (5’-3’) | Reverse primer (5'-3') | A. t. (^o^C) | Size (bp) |
| --- | --- | --- | --- | --- |
| Rpi-vnt1.1 and RB overlap |  |  |  |  |
| Rpi-vnt1.1 RB-F & -R | GCTGCGTTAATTATTTACAT | GTTGGTTGATTACTTGAACT | 55 | 587 |
| RB and Rpi-blb2 overlap |  |  |  |  |
| RB Rpi-blb2-F & -R | AAAAAGAAGATTTCATGCGC | CATTTTGGTTCATGAGTTCA | 55 | 363 |
| *nptII* probe |  |  |  |  |
| NPTII-F & -R | CAGCAATATCACGGGTAGCCA | GGCTATTCGGCTATGACTGGG | 55 | 636 |
| Right (RBd) and left (LBd) border sequences |  |  |  |  |
| Rpi-blb2-RBd-F & -R | TCGTGACTGGGAAAACCCTG | TCTCTTAGGTTTACCCGCCA | 58 | 231 |
| LBd-NPTII-F & -R | TGACGAGTTCTTCTGAGCGG | CAACTTAATAACACATTGCGGACG | 60 | 309 |
| Vector backbone sequences |  |  |  |  |
| 93BB-F & -R | CAAGACGAACTCCAATTCAC | ATATATCCTGCCACCAGC | 60 | 378 |
| 93BB3-F & -R | GTAAGCGGCTGGGTTGTCTG | TCGACAGACGGAAAACGG | 58 | 377 |
| 93BB4-F & -R | ACTTTGATCCAACCCCTCC | AGGTGGTCAAGCATCCTG | 62 | 374 |
| *Avrblb1 effector gene* |  |  |  |  |
| *ipi01*-F & -R | TGAGTACAACGCCGATGAAA | AGATGCTTGGTCCTTAGGATTC | 60 | 300 |
| *ipi02*-F & -R | CGCATCGATGGTTTCATCCAATCTCA | TTTGAATCTGTTGAAAAATACGTTAC | 58 | 390 |
| *ipi03*-F & -R | CGCATCGATGGTTTCATCCAATCTCA | GCTAGCGGAAGCCTTATCGATATA | 61 | 370 |
| *ipi04*-F & -R | CAACACCGCCGGGCATG | CAACACCGTCTTGGACTGAG | 60 | 230 |
| *Avrblb2 effector gene* |  |  |  |  |
| *Avrblb2^Ala69^*-F & -R | AGCTAGGAGCAGCGCCGTC | CTCTTGACGATCTTGTTAGTACTCTGGGC | 61 | 192 |
| *Avrblb2^Ile69^*-F & -R | AGCTAGGAGCAGCGCCGTC | CTTCTTGATGATCTTGTTAGTACTCGGGAT | 61 | 193 |
| *Avrblb2^Phe69^*-F & -R | AGCTAGGAGCAGCGCCGTC | GCTTCTTGATGATCTTGTTAGTACTCGGAAA | 60 | 194 |
| *Avrblb2^Val69^*-F & -R | AGCTAGGAGCAGCGCCGTC | GCTTCTTGACGATCTTGTTAGTACTCTGGAC | 61 | 194 |
| *Avrvnt1 effector gene* |  |  |  |  |
| *Avrvnt1*-F & -R | CGAAGTTGACGGCTCCTG | GGCTCGCTTGAACAAATCC | 60 | 200 |
| *Avr3a effector gene* |  |  |  |  |
| *Avr3a*-F & -R | ATGTGGCTGCGTTGACGGAGA | CCCTGTTGTGCTGCCACCAC | 60 | 120 |

**Table S2:** Transgenic materials selected for field evaluation based on average of leaf damage in whole-plant bioassays in greenhouse resistance tests.

| Events | Leaf damage (%) | Leaf damage class* |
| --- | --- | --- |
| Des.254 | 0 | ND |
| Des.259 | 0 | ND |
| Des.1 | 3 | LD |
| Des.262 | 3 | LD |
| Des.255 | 5 | LD |
| Des.14 | 6 | LD |
| Des.6 | 6 | LD |
| Des.260 | 6 | LD |
| Des.25 | 34 | MD |
| Des.245 | 42 | MD |
| Des.21 | 43 | MD |
| Des.16 | 53 | MD |
| Vic.1 | 0 | ND |

* **ND**: no visible damage on any leaf

**LD**: low damage only small areas affected on few leaves

**MD**: moderately damaged leaves

**Table S3**: Expression of the *Phytophthora infestans* effector genes from isolates collected on late blight infected non-transgenic potato control plants during the mock trial and the subsequent confined field trials. The presence of the effector variants coincides with the lineage detected which were determined by using standard microsatellite genotyping protocols. Each sample is one isolate.

| Sample Source | *R* gene | Cognate effector gene | Effector variant^a^ | Lineage | No. of samples | No. of positive variant |
| --- | --- | --- | --- | --- | --- | --- |
| Mock Trial | *RB* | *Avrblb1* | *ipi01* | US-1 | 12 | 0 |
| Mar-Apr 2014 |  |  | *ipi02* | US-1 | 12 | 0 |
|  |  |  | *ipi03* | US-1 | 12 | 0 |
|  |  |  | *ipi04* | US-1 | 12 | 0 |
|  | *Rpi-blb2* | *Avrblb2* | *Avrblb2^Ala69^* | US-1 | 12 | 12 |
|  |  |  | *Avrblb2^Ile69^* | US-1 | 12 | 12 |
|  |  |  | *Avrblb2^Phe69^* | US-1 | 12 | 12 |
|  |  |  | *Avrblb2^Val69^* | US-1 | 12 | 12 |
|  | *Rpi-vnt1.1* | *Avrvnt1* | *Avrvnt1* | US-1 | 12 | 12 |
| CFT-1 | *RB* | *Avrblb1* | *ipi01* | 2_A1/US-1 | 52 | 42 |
| Jun-Sep 2015 |  |  | *ipi02* | 2_A1/US-1 | 52 | 42 |
|  |  |  | *ipi03* | 2_A1/US-1 | 52 | 0 |
|  |  |  | *ipi04* | 2_A1/US-1 | 52 | 0 |
|  | *Rpi-blb2* | *Avrblb2* | *Avrblb2^Ala69^* | 2_A1/US-1 | 52 | 52 |
|  |  |  | *Avrblb2^Ile69^* | 2_A1/US-1 | 52 | 52 |
|  |  |  | *Avrblb2^Phe69^* | 2_A1/US-1 | 52 | 10 |
|  |  |  | *Avrblb2^Val69^* | 2_A1/US-1 | 52 | 52 |
|  | *Rpi-vnt1.1* | *Avrvnt1* | *Avrvnt1* | 2_A1/US-1 | 52 | 52 |
| CFT-2 | *RB* | *Avrblb1* | *ipi01* | 2_A1/US-1 | 27 | 24 |
| Oct-Jan 2016 |  |  | *ipi02* | 2_A1/US-1 | 27 | 24 |
|  |  |  | *ipi03* | 2_A1/US-1 | 27 | 0 |
|  |  |  | *ipi04* | 2_A1/US-1 | 27 | 0 |
|  | *Rpi-blb2* | *Avrblb2* | *Avrblb2^Ala69^* | 2_A1/US-1 | 27 | 27 |
|  |  |  | *Avrblb2^Ile69^* | 2_A1/US-1 | 27 | 27 |
|  |  |  | *Avrblb2^Phe69^* | 2_A1/US-1 | 27 | 9 |
|  |  |  | *Avrblb2^Val69^* | 2_A1/US-1 | 27 | 27 |
|  | *Rpi-vnt1.1* | *Avrvnt1* | *Avrvnt1* | 2_A1/US-1 | 27 | 27 |
| CFT-3 | *RB* | *Avrblb1* | *ipi01* | 2_A1 | 14 | 14 |
| Mar-Jun 2016 |  |  | *ipi02* | 2_A1 | 14 | 14 |
|  |  |  | *ipi03* | 2_A1 | 14 | 0 |
|  |  |  | *ipi04* | 2_A1 | 14 | 0 |
|  | *Rpi-blb2* | *Avrblb2* | *Avrblb2^Ala69^* | 2_A1 | 14 | 14 |
|  |  |  | *Avrblb2^Ile69^* | 2_A1 | 14 | 14 |
|  |  |  | *Avrblb2^Phe69^* | 2_A1 | 14 | 14 |
|  |  |  | *Avrblb2^Val69^* | 2_A1 | 14 | 11 |
|  | *Rpi-vnt1.1* | *Avrvnt1* | *Avrvnt1* | 2_A1 | 14 | 14 |
| CFT-4 | *RB* | *Avrblb1* | *ipi01* | 2_A1 | 25 | 25 |
| Oct-Feb 2017 |  |  | *ipi02* | 2_A1 | 25 | 25 |
|  |  |  | *ipi03* | 2_A1 | 25 | 0 |
|  |  |  | *ipi04* | 2_A1 | 25 | 0 |
|  | *Rpi-blb2* | *Avrblb2* | *Avrblb2^Ala69^* | 2_A1 | 25 | 25 |
|  |  |  | *Avrblb2^Ile69^* | 2_A1 | 25 | 25 |
|  |  |  | *Avrblb2^Phe69^* | 2_A1 | 25 | 24 |
|  |  |  | *Avrblb2^Val69^* | 2_A1 | 25 | 25 |
|  | *Rpi-vnt1.1* | *Avrvnt1* | *Avrvnt1* | 2_A1 | 25 | 25 |

^a^ following Vleeshouwers et al. 2011

**Table S4:** Morphology of tubers harvested from transgenic events and their control varieties from two seasons (CFT-2 and CFT-3).

| Plant materials | CFT-2 | | | CFT-3 | | |
| --- | --- | --- | --- | --- | --- | --- |
|  | **Tuber skin color** | **Tuber flesh color** | **Tuber shape** | **Tuber Skin color** | **Tuber flesh color** | **Tuber shape** |
| Des.1 | Red | Light cream | Oblong | Red | Light cream | Oblong |
| Des.6 | Red | Cream | Oblong | Red | Light cream | Oblong |
| Des.14 | Red | Light cream | Oblong | Red | Light cream | Oblong |
| Des.16 | Red | White | Oblong | Red | Light cream | Oblong |
| Des.21 | Red | Light cream | Oblong | Red | Light cream | Oblong |
| Des.25 | Red | Light cream | Oblong | Red | Light cream | Oblong |
| Des.245 | Red | Light cream | Oblong | Red | Light cream | Oblong |
| Des.249 | Red | Light cream | Oblong | Red | Light cream | Oblong |
| Des.254 | Red | Light cream | Oblong | Red | Light cream | Oblong |
| Des.255 | Red | Light cream | Oblong | Red | Light cream | Oblong |
| Des.259 | Red | Cream | Oblong | Red | Light cream | Oblong |
| Des.260 | Red | Light cream | Oblong | Red | Light cream | Oblong |
| Des.262 | Red | Light cream | Oblong | Red | Light cream | Oblong |
| Desiree | Red | Light cream | Oblong | Red | Light cream | Oblong |
| Vic.1 | Red | White | Round | Light red | White | Round |
| Victoria | Light red | White | Round | Light red | White | Round |
| Cruza 148 | White | White with purple ring | Round | White | White with purple ring | Round |
